# Open-case series of a remote administration and group setting comprehensive behavioral intervention for tics (RG-CBIT): A pilot trial

**DOI:** 10.3389/fpsyt.2022.890866

**Published:** 2022-07-26

**Authors:** Takeshi Inoue, Kohei Togashi, Jumpei Iwanami, Douglas W. Woods, Ryoichi Sakuta

**Affiliations:** ^1^Dokkyo Medical University Saitama Medical Center, Saitama, Japan; ^2^Department of Psychology, Marquette University, Milwaukee, WI, United States

**Keywords:** tic disorders, Tourette syndrome, the comprehensive behavioral intervention for tics (CBIT), group, remote, telehealth

## Abstract

**Purpose:**

The comprehensive behavioral intervention for tics (CBIT) is the first-line psychotherapeutic treatment for individuals with tic disorders. However, most patients with tic disorders do not have access to CBIT due to different factors including lack of trained therapists, treatment cost, and travel distance. Such barriers are more prominent in non-English speaking countries. Therefore, the current study assessed the preliminary efficacy, feasibility, and acceptability of remotely administered group CBIT (RG-CBIT) in Japan.

**Methods:**

This was an open-case series that adopted the AB design. Three Japanese children aged between 6 and 13 years who were diagnosed with TS were recruited. RG-CBIT was developed based on the published CBIT manual. Videoconference application, slide presentation software, and cloud learning platform were used as appropriate.

**Results:**

The Yale Global Tic Severity Scale scores of all participants decreased from baseline to post-treatment. That is, the score reduced by an average of 7.0. Regarding feasibility and acceptability, the attendance rate of participants was 100%, and the process measurement items had favorable scores.

**Conclusions:**

RG-CBIT had satisfactory efficacy, feasibility, and acceptability. Hence, it could mitigate the barriers for treatment access.

## Introduction

Tics are sudden, repetitive, non-rhythmic movements (i.e., motor tics) or vocalizations (i.e., vocal tics). Tic disorder is one of the neurodevelopmental disorders characterized by motor and/or vocal tics that begin in childhood. Tics may persist, and the type of tics can change over time. Symptom severity commonly peaks at the early years of teenage life (1). Chronic tic disorder (CTD) is characterized by tics lasting more than 1 year, and tics may be either motor or vocal, but not both. Tourette’s disorder (TD), also known as Tourette syndrome (TS), is characterized by the presence of one or more chronic multiple motor and vocal tics (2). In a previous meta-analysis, the prevalence of TS was 0.77%, and TS is more common in boys (3).

**FIGURE 1:**
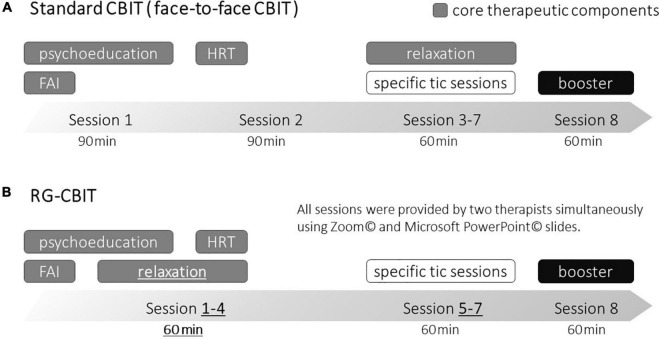
Difference between RG-CBIT and standard CBIT. *FAI*, functional assessments and interventions; *HRT*, habit reversal training; *RG-CBIT*, remotely administered group comprehensive behavioral intervention for tics; *CBIT*, comprehensive behavioral intervention for tics. The upper part **(A)** is a schematic diagram of the standard CBIT, and the lower part **(B)** is a schematic diagram of RG-CBIT. The dark gray cells indicate core therapeutic components. There were three primary differences between RG-CBIT and the standard CBIT (underline and bold text). First, the duration of all sessions were changed to 60 min. Second, the learning relaxation components were moved to the first session. Third, the core therapeutic components were trained in the first four sessions (combined 240 min) rather than the first two sessions (combined 180 min).

In most cases, tic disorders are mild to moderate, and they do not always require treatment. However, if tics are severe or children experience several psychosocial problems, such as deteriorating relationships with family and friends and interference with school and extracurricular activities, then treatment is required (4). The comprehensive behavioral intervention for tics (CBIT) is the first-line treatment for individuals with tic disorders (5). Woods and colleagues developed CBIT (6), which includes the core therapeutic components of psychoeducation, functional assessments and interventions (FAI), habit reversal training (HRT), and relaxation training (Figure 1). CBIT was designed to include eight sessions weekly for 10 weeks, followed by periodic booster session(s) to maintain treatment gains and to learn how to deal with tics that may emerge in the future. The first two sessions last 90 min (combined 180 min), during which patients and their parents receive psychoeducation about tics and learn the basics of the functional assessments/interventions and HRT procedures. The remaining sessions last 60 min and focus on administering core therapeutic components to additional tics and teaching patients and their parents regarding relaxation skills. CBIT was initially tested among children aged 9 years and older. However, a recent study showed that CBIT is effective in young children aged between 5 and 8 years, incorporating enjoyable and ingenious elements of the game called “the opposite game” (7). In this study, the authors highlighted that involving parents in behavioral interventions for young children improves the acceptability, efficacy, and durability.

A controlled clinical study has shown that CBIT, similar to pharmacologic treatment, can improve tics in children and adults without causing significant side effects (8–10). Although CBIT is effective, several children with CTD and TS cannot access CBIT because of several factors including lack of trained therapists, treatment cost, travel distance, and time commitment (11–13). CBIT is not widely available particularly in non-English speaking countries (14). To address these barriers and to promote CBIT dissemination, controlled trials of remote administration, such as telehealth and internet-delivered psychotherapy, have been carried out recently. Himle et al. conducted a small randomized controlled trial (RCT) comparing CBIT delivered using the videoconference system and traditional face-to-face CBIT (15). Results showed that both formats were equally beneficial to children with tic disorders. In addition to CBIT, a large long-term follow-up study of ERP is underway in the United Kingdom. Remote Administration is an emerging and significant topic in behavioral therapy (16).

Group CBIT is another method that can increase treatment accessibility. Zimmerman-Brenner et al. conducted an RCT of group CBIT and group educational intervention. Results showed that group CBIT significantly decreased total and motor tic severity (17).

However, CBIT is not widely available in Japan due to a considerable lack of well-trained therapists. Although there is a Japanese translation of the manual established by Woods et al. (6), which was published in 2018, training opportunities for learning CBIT procedures are limited among therapists. Moreover, CBIT is not covered by public health insurance in Japan; thus, the cost burden on patients is substantial. Therefore, the current study aimed to assess the preliminary efficacy, feasibility, and acceptability of remotely administered group CBIT (RG-CBIT) for reducing tics in children with TS *via* an open-case series.

## Materials and methods

### Study design and ethical considerations

This was an open-case series that utilized the AB design study (two-phase design comprising a baseline and an intervention phase). The recruitment phase was 4 weeks; the baseline phase, 10 weeks; and the intervention phase, 10 weeks. Clinical assessments were performed 5 days before baseline (Ax1 assessment 1) and the first session (Ax2 assessment 2), and 5 days after the end of the sessions (Ax3 assessment 3). Standard care, including medication treatment, was continued (not changed) throughout the study period.

Written informed consents were obtained from the participants. This study was approved by the Ethical Committee of Dokkyo Medical University Saitama Medical Center (21019).

### Participants

The inclusion criteria were as follows: (a) individuals aged between 6 and 15 years, (b) those with a diagnosis of TS based on the DSM-5 criteria (18), (c) those with a score of ≥ 14 for the total tic severity score on the Yale Global Tic Severity Scale (YGTSS), and (d) those who are medication free or on a stable medication for the treatment of tics, obsessive-compulsive disorder (OCD), and attention deficit hyperactivity disorder (ADHD) for at least 6 weeks, without planned changes during the study period. The exclusion criteria were as follows: (a) individuals with a diagnosis of other psychiatric disorders except for TS, ADHD, and OCD based on the DSM-5 criteria, (b) those with any serious physical disease, psychosocial, or neurological condition requiring treatment, (c) those with previous behavioral therapy for TS, and (d) those with lack of accessible home computer or tablet device and/or high-speed internet connection.

Three Japanese children aged between 6 and 13 years and diagnosed with TS were recruited from Child Development and Psychosomatic Medicine Center, Dokkyo Medical University Saitama Medical Center, in February 2021. The clinical characteristics were obtained during the recruitment phase (as shown in Table 1).

**TABLE 1:** Clinical characteristic details.

	Age	Sex	Diagnosis	Age of TS onset	Comorbidity	Medication	ADHD RS-IV	CY-BOCS
Case 1	6	M	TS	4	ADHD	-	22	0
Case 2	9	M	TS	6	ADHD	Guanfacine 3 mg/d	25	0
Case 3	13	F	TS	4	ADHD OCD	Guanfacine 4 mg/d Atomoxetine 50 mg/d	25	11

TS, Tourette syndrome; ADHD RS-IV, attention deficit hyperactivity disorder rating scale IV; CY-BOCS, children’s Yale-Brown obsessive compulsive scale.

### Materials

Zoom© (Zoom Video Communications, Inc.), a secure, reliable video platform, was adopted to communicate between participants and therapists. It was chosen because of its high image resolution, availability, and affordability in the general population in Japan. The breakout-room function was another important factor that contributed to the decision to use Zoom©. Microsoft PowerPoint© is a slide presentation software that was used to explain the CBIT session contents. Google docs© and sheets© were used to design homework materials, and Google Classroom© is a cloud-based learning platform used to assign and submit the homework and provide feedback for the homework. Participants and therapists used their home computer or tablet with high-speed internet connection, and a built-in webcam was used to monitor the participant’s movements, positioning, and other non-vocal responses (e.g., nodding or raising the hand to indicate that a task is completed) during the meetings. Zoom, or email or cellular phones were used to communicate with participants outside of the RG-CBIT sessions as needed.

### RG-CBIT and procedure

RG-CBIT was developed based on the CBIT manual developed by Woods and colleagues in terms of the number and components of treatment sessions, distribution of CBIT contents, and length of intervention. RG-CBIT was modified by the authors [Takeshi Inoue (TI) who has a Ph.D. degree in Medicine and a Board-Certified Member of the Japanese Society of Child Neurology and who is well experienced in all aspects of TS and CTD, Kohei Togashi (KT) who was a clinical psychologist certified in Japan and a doctoral-level behavior analyst, and Jumpei Iwanami (JI) who was a clinical psychologist certified in Japan and has a master’s degree in psychology]. Consultation with Dr. Douglas Woods was performed as needed. There were three primary differences between RG-CBIT and standard CBIT. First, the duration of all sessions was changed to 60 min because duration of 90 min remote session was lengthy for young children. Second, learning relaxation components were moved to the first session since they were easy to teach and perform. Third, the skills required to implement these therapeutic components are complex and we wanted to provide the participants with multiple opportunities to practice them. Thus, core therapeutic components (psychoeducation, FAI, HRT, and relaxation training) were trained in the first four sessions (combined 240 min) rather than the first two sessions (combined 180 min) (Figure 1).

All sessions were facilitated simultaneously by the two qualified clinical psychologists in one group. Educational slides were basically adopted strictly from the handbook. The slides were designed familiar and ingenious including illustrations, videos, and quizzes, to be enjoyable and approachable for even young children. The video contents included scenes about tic maintained by attention (social positive reinforcement), escape/avoidance (social negative reinforcement), and automatic reinforcement. KT was in charge of administering the core therapeutic components, and JI provided technical support as needed during the first four sessions (core therapeutic sessions). A lecture on social support, or how to give appropriate praise and reminder for the competing response (a specific action that makes the tic more difficult to emerge), was given at the end of the core therapeutic sessions to parents. Besides, the parents were involved throughout the sessions. For instance, helping the children with selecting appropriate competing responses, conducting homework with the children, and monitoring and recording the children’s tics.

In sessions 5–7, which focused on a specific individual tic (specific tic sessions), HRT was provided in group settings according to each participant’s tic. If one participant (child–parent dyads) participated in the HRT, other participants observed the training. FAI was conducted separately (one on one) using the breakout-room function of Zoom© with KT. We created opportunities for participants to talk with the therapist individually. Hence, they could discuss issues that they may not be comfortable sharing with the group. A booster session was provided to review the topics covered in the previous sessions and treatment gains, and to learn how to deal with newly emerging tics in the future (as shown in Figure 1).

The core therapeutic sessions and specific tic sessions (the first seven sessions) were held weekly. A booster session was held 4 weeks after the last specific tic session to promote skill maintenance. The duration of the entire treatment program was 10 weeks. Consistent with the manual, a weekly homework was created using Google docs© and sheets©, which was assigned and submitted *via* Google Classroom©. KT checked and provided feedback weekly and individually *via* Google Classroom©.

### Assessment measures

Clinical assessments were performed 5 days before the baseline (Ax1 assessment 1) and the first session (Ax2 assessment 2), and 5 days after the end of the sessions (Ax3 assessment 3). Table 2 depicts the detailed assessment schedule. Questionnaire-based assessments were mailed to the participants, and interview-based assessments were conducted by an experienced medical doctor (non-therapist) *via* Zoom©.

**TABLE 2:** Schedule of assessments.

	Ax 1	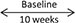	Ax 2	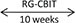	Ax 3
YGTSS	x		x		x
CGI-S	x		x		x
CGI-I			x		x
PUTS	x		x		x
SDQ	x		x		x
CSQ-8J					x
J-WAI-SR					x
Modified-TEI					x

Ax, assessment; YGTSS, Yale Global Tic Severity Scale; CGI-S, The Clinical Global Impression-severity score; CGI-I, The Clinical Global Impression-Improvement scale; PUTS, The Premonitory Urge of Tics Scale; SDQ, The Strength and Difficulties Questionnaire; CSQ-8J, The Japanese version of the Client Satisfaction Questionnaire-8; J-WAI-SR, The Japanese version of the Working Alliance Inventory-Short Revised; Modified-TEI, Modified version of the Treatment Evaluation Inventory.

#### Yale global tic severity scale (19)

The YGTSS result was the primary outcome measure for evaluating the preliminary efficacy of the intervention for reducing tics. YGTSS is a semi-structured interview that is the gold standard for tic assessment. It yields two separate 0–50-point scales. The Total Tic Severity scale can be used to assess the severity of motor and vocal tic symptoms across the domains of tic number, frequency, intensity, complexity, and interference. The Tic Impairment scale assesses the extent to which the tics lead to impairment in the child’s daily life and activities (impairment scale score: 0–50). In both scales, higher scores indicate more severe tic symptoms or impairment.

#### Clinical global impression (20)

The Clinical Global Impression (CGI) rating scale is one of the most widely used assessment scales for assessing symptom severity and treatment response in intervention studies of patients with mental disorders. The CGI Severity score (CGI-S) is an observer-rated seven-point scale for evaluating illness severity at the time of assessment (scored between 1: normal, not at all ill and 7: among the most extremely ill patients). The seven-point CGI Improvement scale (CGI-I) rates improvement from 1 (very much improved) and 7 (very much worse due to intervention). A rating of 4 indicates that a patient did not experience any improvement after the intervention.

#### Premonitory urge of tics scale (21, 22)

Premonitory Urge of Tics Scale (PUTS) is a 9-item self-reported questionnaire scored from 1 to 4 (with a total score of 9–36), which is commonly used to assess premonitory urge strength. The Japanese version was designed using rigid methods, including translation and back translation, and with sufficient internal and concurrent validity.

#### Strength and difficulties questionnaire (23, 24)

Strength and Difficulties Questionnaire (SDQ) is a 25-item questionnaire that is used to assess the emotional and behavioral perspective of children. It was answered by the parents in this study. These items comprise five scales, which are as follows: emotional symptoms, conduct problems, hyperactivity/inattention, peer relationships problem, and prosocial behavior. The previous four subscales were added together to generate the total difficulty score (range: 0–40) (based on 20 items), with higher scores indicating more severe conditions.

#### Japanese version of the client satisfaction questionnaire-8 (25, 26)

Client Satisfaction Questionnaire-8 (CSQ-8) is an 8-item self-report questionnaire providing comprehensive measures of patient or client satisfaction with services in the medical and mental health primary care. Each item was scored from 1 to 4 (with a total score of 8–32), with higher scores representing higher satisfaction.

#### Japanese version of the working alliance inventory-short revised (27, 28)

Working Alliance Inventory-Short Revised (WAI-SR) is a recently refined self-reported questionnaire evaluating the therapeutic alliance that assesses three key aspects: agreement on the tasks of therapy, agreement on the goals of therapy, and development of an affective bond. It contains 12 questions according to the 7-point Likert scale (with a total score of 12–84), with higher scores indicating good alliance.

#### Modified version of the treatment evaluation inventory (29, 30)

Treatment Evaluation Inventory (TEI) is a commonly used measure of treatment acceptability. Modified TEI contains 11 items divided into two subscales: (a) general acceptability scale (8 items) and (b) negative aspect subscale (3 items). Each item is seven-point Likert scale (with a total score of 11–77). A score of 44 indicates moderately favorable attitudes toward the treatment, with higher scores representing favorable treatment.

## Results

Three participants attended all the sessions and completed all assessments.

**FIGURE 2:**
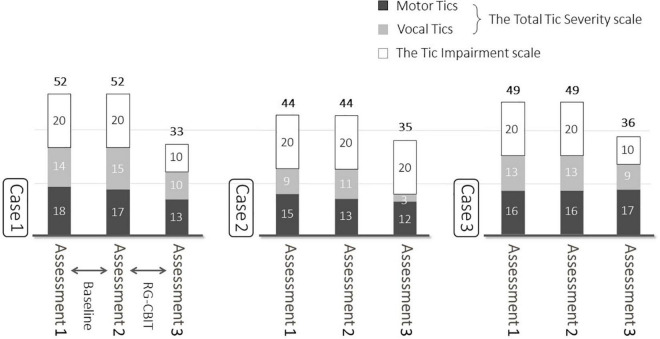
Baseline and post-treatment YGTSS. At baseline, the Total Tic Severity scale score did not change. However, after the intervention, those scores decreased by 9, 9, and 3 points for cases 1, 2, and 3, respectively. The score reduced by an average of 7.0.

Figure 2 shows the YGTSS scores. During baseline (between Ax1 and Ax 2), the Total Tic Severity scale score did not change. However, after the intervention, the scores decreased by 9, 9, and 3 points for cases 1, 2, and 3, respectively. The score reduced by an average of 7.0. The Tic Impairment scale score did not change at baseline. However, it decreased by 10, 0, and 10 points for cases 1, 2, and 3, respectively, after the intervention.

**FIGURE 3:**
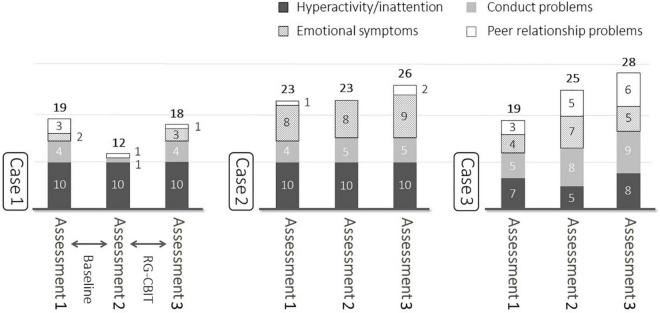
Baseline and post-treatment SDQ. After the intervention, the total SDQ scores increased by 6, 3, and 3 points for cases 1, 2, and 3, respectively.

Table 3 depicts the CGI and PUTS scores. The CGI-S and CGI-I score showed no change or worsened at baseline. Nevertheless, they showed improvement in two cases after the intervention (Table 3). Figure 3 shows the SDQ scores. After the intervention, the total SDQ scores increased by 6, 3, and 3 points for cases, respectively.

**TABLE 3:** Clinical outcomes (CGI and PUTS).

		Ax 1	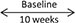	Ax 2	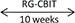	Ax 3
CGI-S						
	Case 1	5		5		3
	Case 2	4		4		4
	Case 3	3		5		4
CGI-I						
	Case 1	NA		4		2
	Case 2	NA		4		4
	Case 3	NA		5		3
PUTS						
	Case 1	20		13		29
	Case 2	24		26		23
	Case 3	21		21		19

Ax, Assessment; NA, not available; CGI-S, The Clinical Global Impression-severity score; CGI-I, The Clinical Global Impression-Improvement scale; PUTS, The Premonitory Urge of Tics Scale.

Table 4 shows the process measures (CSQ-8J, J-WAI-SR, and Modified-TEI). The average CSQ-8J, J-WAI-SR, and Modified-TEI were 28.0, 75.0, and 65.3, respectively.

**TABLE 4:** Process measures (CSQ-8J, J-WAI-SR and TEI-R).

Measure	Scores range	Case 1	Case 2	Case 3	Average
CSQ-8J	8–32	29	30	25	28.0
J-WAI-SR	12–84	84	75	66	75.0
Modified-TEI	11–77	67	69	60	65.3

CSQ-8J, The Japanese version of the Client Satisfaction Questionnaire-8; J WAI-SR, The Japanese version of the Working Alliance Inventory-Short Revised; Modified-TEI, Modified version of the Treatment Evaluation Inventory.

## Discussion

Using the Bayesian network meta-analysis methods, Liang et al. showed that CBIT is an effective treatment for patients with TS (31). However, most children and adolescents with TS, particularly in non-English speaking countries, do not have access to CBIT because of several barriers. Thus, we developed RG-CBIT to eliminate barriers such as lack of trained therapists, treatment cost, and travel distance. The current study aimed to evaluate the preliminary efficacy, feasibility, and acceptability of RG-CBIT *via* an open case series.

Tic severity and impairment reduced from baseline to post-intervention in this study. Tic severity and tic-related impairment reduced based on the assessment using YGTSS. Piacentini et al. performed a large RCT examining the efficacy of individual face-to-face CBIT. Results showed that the total tic severity score decreased by an average of 7.6 points (8). The current study showed a similar improvement. That is, the total tic severity score decreased by 7.0 points even though remote administration and group format were applied. A 6-7 point decrease in the total tic severity score is an indicator of treatment response (32), and we believe that our trial was clinically effective. Impairment scores on YGTSS were not high from baseline for all 3 participants, this is probably because they had previously attended our facility, received psycho-education as usual medical care, and consulted with the school.

Moreover, improvement was also observed based on CGI, and this finding supports the efficacy of RG-CBIT. Even though the number of sessions specifically focused on tics, it was less than that specified in the original CBIT manual (Figure 1), and the results of the current study were comparable to that of previous ones. One potential explanation is that HRT was provided in groups. Thus, the participants could also learn to deal with the tic symptoms of other patients. Another reason is that the four separate core therapeutic components sessions may have a positive impact on the retention of knowledge and skills.

Despite the explicit teaching about awareness to the perception of premonitory urges and instruction of voluntary competing response in HRT contents, the PUTS score did not improve with RG-CBIT. Previous studies have reported similar results (8, 10, 33).

Strength and difficulties questionnaire was used to assess the impact of RG-CBIT on the QOL of patients. Results showed that the QOL increased after the intervention. SDQ is affected by different factors including school life and family relationships. Thus, it might have been influenced by other factors other than the intervention in this study (23, 34).

Regarding the feasibility and acceptability of RG-CBIT, the attendance rate of participants was 100%, and the patients had strong treatment satisfaction and therapeutic alliance based on the process measures (CSQ-8J, J-WAI-SR, and TEI-R) (Table 4). The CSQ-8J and J-WAI-SR findings were similar to those reported by Ricketts et al. (35) and Himle et al. (15). This finding is particularly significant, as doing group remote therapy has become even more important during the coronavirus disease 2019 pandemic. The average scores were favorable. However, these scores were lower in case 3. In this open case series, the participants were aged 6, 9, and 13 years. Case 3 was the oldest among the three participants and was only a junior high school student. Group sessions were conducted using methods that can help the youngest participant understand instructions and maintain attention during the session, therefore, 13 years-girl may have felt a little bored. The age difference might have affected the process measures of case 3, and the inclusion of matching age groups may enhance the acceptability of this treatment. Additionally, homework wasn’t submitted in Case 3, occasionally. It is also important to devise ways to enhance the submission of homework.

Regarding materials, Zoom© was a reliable video platform. We can observe their facial expression and fine motor tics even eye blinking, lip cramp, and so on. The breakout-room was useful for private consultations in FAI as intended, except that it is a bit complicated to operate. The participants were asked to keep their video cameras on throughout the sessions so the therapist could monitor their responses. As for Google Classroom©, it was a favorable way to provide feedback for the homework, however, trouble occurred occasionally with sharing files between participants and the therapist. The current study had several limitations. That is, the research design was not controlled, and a small sample size was included. Moreover, RG-CBIT was delivered during the circumstances of a COVID-19 pandemic. The repeated lockdowns and restrictions on school life might have affected the mental health of all children, and may have had no small impact on the participants in this study. Finally, follow-up assessment was not conducted. Thus, whether gains were maintained is unknown. In the near future, we plan to conduct an assessor-blind RCT of RG-CBIT on children with tic disorders that can address these limitations.

In conclusion, RG-CBIT had satisfactory efficacy and adequate feasibility and acceptability. Although further studies are required, the current research supported previous notions showing that RG-CBIT is effective for reducing tic severity and impairment. Moreover, remote administration and group setting could mitigate barriers for accessing CBIT such as lack of experienced psychotherapists, treatment cost, and travel distance.

## Data availability statement

The original contributions presented in this study are included in the article/supplementary material, further inquiries can be directed to the corresponding author.

## Ethics statement

The studies involving human participants were reviewed and approved by the ethical committee of Dokkyo Medical University Saitama Medical Center (21019). Written informed consent to participate in this study was provided by the participants’ legal guardian/next of kin.

## Author contributions

TI, KT, and JI collected patient’s data. KT and JI facilitated the sessions. TI, KT, and DW compiled the manuscript. TI, KT, and RS participated in the design of this study. DW and RS supervised this research. All authors reviewed the manuscript and approved it in its current form and have approved the ordering of authorship.

## References

[1] Leckman JF Zhang H Vitale A Lahnin F Lynch K Bondi C Course of tic severity in tourette syndrome: the first two decades. *Pediatrics.* (1998) 102:14–9. 10.1542/PEDS.102.1.14 9651407

[2] Ueda K Black KJ. A comprehensive review of tic disorders in children. *J Clin Med.* (2021) 10:1–32. 10.3390/jcm10112479 34204991 PMC8199885

[3] Knight T Steeves T Day L Lowerison M Jette N Pringsheim T. Prevalence of tic disorders: a systematic review and meta-analysis. *Pediatr Neurol.* (2012) 47:77–90. 10.1016/j.pediatrneurol.2012.05.002 22759682

[4] Espil FM Capriotti MR Conelea CA Woods DW. The role of parental perceptions of tic frequency and intensity in predicting tic-related functional impairment in youth with chronic tic disorders. *Child Psychiatry Hum Dev.* (2014) 45:657–65. 10.1007/S10578-013-0434-2 24395287 PMC4085134

[5] Pringsheim T Okun MS Müller-Vahl K Martino D Jankovic J Cavanna AE Practice guideline recommendations summary: treatment of tics in people with tourette syndrome and chronic tic disorders. *Neurology.* (2019) 92:896–906. 10.1212/WNL.0000000000007466 31061208 PMC6537133

[6] Woods DW Piacentini J Chang S Deckersbach T Ginsburg G Peterson A *Managing Tourette Syndrome - A Behavioral Intervention for Children and adults Therapist Guide.* New York, NY: Oxford University Press (2008).

[7] Bennett SM Capriotti M Bauer C Chang S Keller AE Walkup J Development and open trial of a psychosocial intervention for young children with chronic tics: the CBIT-JR study. *Behav Ther.* (2020) 51:659–69. 10.1016/j.beth.2019.10.004 32586437

[8] Piacentini J Woods DW Scahill L Wilhelm S Peterson AL Chang S Behavior therapy for children with tourette disorder: a randomized controlled trial. *JAMA.* (2010) 303:1929–37. 10.1001/jama.2010.607 20483969 PMC2993317

[9] Wilhelm S Peterson AL Piacentini J Woods DW Deckersbach T Sukhodolsky DG Randomized trial of behavior therapy for adults with tourette syndrome. *Arch Gen Psychiatry.* (2012) 69:795–803. 10.1001/archgenpsychiatry.2011.1528 22868933 PMC3772729

[10] Sukhodolsky DG Woods DW Piacentini J Wilhelm S Peterson AL Katsovich L Moderators and predictors of response to behavior therapy for tics in tourette syndrome. *Neurology.* (2017) 88:1029–36. 10.1212/WNL.0000000000003710 28202705 PMC5384839

[11] Ricketts EJ Bauer CC Ran D Himle MB Woods DW. Pilot open case series of voice over internet protocol-delivered assessment and behavior therapy for chronic tic disorders. *Cogn Behav Pract.* (2016) 23:40–50. 10.1016/j.cbpra.2014.09.003 30595642 PMC6309412

[12] Jakubovski E Reichert C Karch A Buddensiek N Breuer D Müller-Vahl K. The ONLINE-TICS study protocol: a randomized observer-blind clinical trial to demonstrate the efficacy and safety of internet-delivered behavioral treatment for adults with chronic tic disorders. *Front Psychiatry.* (2016) 7:119. 10.3389/fpsyt.2016.00119 27445874 PMC4928510

[13] Rachamim L Zimmerman-Brenner S Rachamim O Mualem H Zingboim N Rotstein M. Internet-based guided self-help comprehensive behavioral intervention for tics (ICBIT) for youth with tic disorders: a feasibility and effectiveness study with 6 month-follow-up. *Eur Child Adolesc Psychiatry.* (2020) 31:275–87. 10.1007/S00787-020-01686-2 33231786 PMC7683326

[14] Chen CW Wang HS Chang HJ Hsueh CW. Effectiveness of a modified comprehensive behavioral intervention for tics for children and adolescents with tourette’s syndrome: a randomized controlled trial. *J Adv Nurs.* (2020) 76:903–15. 10.1111/jan.14279 31782167

[15] Himle MB Freitag M Walther M Franklin SA Ely L Woods DW. A randomized pilot trial comparing videoconference versus face-to-face delivery of behavior therapy for childhood tic disorders. *Behav Res Ther.* (2012) 50:565–70. 10.1016/j.brat.2012.05.009 22743661

[16] Hollis C Hall CL Jones R Marston L Novere ML Hunter R Therapist-supported online remote behavioural intervention for tics in children and adolescents in England (ORBIT): a multicentre, parallel group, single-blind, randomised controlled trial. *Lancet Psychiatry.* (2021) 8:871–82. 10.1016/S2215-0366(21)00235-2 34480868 PMC8460453

[17] Zimmerman-Brenner S Pilowsky-Peleg T Rachamim L Ben-Zvi A Gur N Murphy T Group behavioral interventions for tics and comorbid symptoms in children with chronic tic disorders. *Eur Child Adolesc Psychiatry.* (2021) 31:637–48. 10.1007/S00787-020-01702-5 33415472

[18] American Psyciatric Publishing. *Diagnostic and Statistical Manual of Mental Disorders.* 5th ed. Washington, D.C: American Psyciatric Publishing (2013).

[19] Leckman JF Riddle MA Hardin MT Ort SI Swartz KL Stevenson J The yale global tic severity scale: initial testing of a clinician-rated scale of tic severity. *J Am Acad Child Adolesc Psychiatry.* (1989) 28:566–73. 10.1097/00004583-198907000-00015 2768151

[20] Berk M Ng F Dodd S Callaly T Campbell S Bernardo M The validity of the CGI severity and improvement scales as measures of clinical effectiveness suitable for routine clinical use. *J Eval Clin Pract.* (2008) 14:979–83. 10.1111/J.1365-2753.2007.00921.X 18462279

[21] Woods DW Piacentini J Himle MB Chang S. Premonitory urge for tics scale (PUTS): initial psychometric results and examination of the premonitory urge phenomenon in youths with Tic disorders. *J Dev Behav Pediatr.* (2005) 26:397–403. 10.1097/00004703-200512000-00001 16344654

[22] Matsuda N Nonaka M Kono T Fujio M Nobuyoshi M Kano Y. Premonitory awareness facilitates tic suppression: subscales of the premonitory urge for tics scale and a new self-report questionnaire for tic-associated sensations. *Front psychiatry.* (2020) 11:592. 10.3389/FPSYT.2020.00592 32719621 PMC7350852

[23] Goodman R. Psychometric properties of the strengths and difficulties questionnaire. *J Am Acad Child Adolesc Psychiatry.* (2001) 40:1337–45. 10.1097/00004583-200111000-00015 11699809

[24] Matsuishi T Nagano M Araki Y Tanaka Y Iwasaki M Yamashita Y Scale properties of the japanese version of the strengths and difficulties questionnaire (SDQ): a study of infant and school children in community samples. *Brain Dev.* (2008) 30:410–5. 10.1016/J.BRAINDEV.2007.12.003 18226867

[25] Nguyen TD Attkisson CC Stegner BL. Assessment of patient satisfaction: development and refinement of a service evaluation questionnaire. *Eval Program Plann.* (1983) 6:299–313. 10.1016/0149-7189(83)90010-1 10267258

[26] Tachimori H Ito H. Nihongoban Client Satisfaction Questionnaire 8- koumokuban no shinraisei oyobi datousei no kentou. *Seishin Igaku.* (1999) 41:711–7.

[27] Hatcher RL Gillaspy JA. Development and validation of a revised short version of the working alliance inventory. *Psychother Res.* (2006) 16:12–25. 10.1080/10503300500352500

[28] Kawamura A Irie T Takebayashi Y Sekiguchi M Iwano S Motoya R Revised short version of working alliance inventory. *Japanese J Behav Cogn Ther.* (2020) 46:191–202. 10.24468/jjbct.20-002

[29] Landreville P Guérette A. Psychometric properties of a modified version of the treatment evaluation inventory for assessing the acceptability of treatments for geriatric depression*. *Can J Aging.* (1998) 17:414–24. 10.1017/S071498080001268X

[30] Oishi A. Which factors enhance willingness to engage in expressive writing? *Japanese J Res Emot.* (2020) 28:1–10. 10.4092/jsre.28.1_1

[31] Liang J h Zhang SX Chen YC Tan KY Zhang JS Zhao Y Role of psychotherapy strategy for the management of patients with tourette syndrome - a bayesian network meta-analysis. *J Psychiatr Res.* (2021) 143:451–61. 10.1016/J.JPSYCHIRES.2021.07.051 34482986

[32] Storch EA De Nadai AS Lewin AB McGuire JF Jones AM Mutch PJ Defining treatment response in pediatric tic disorders: a signal detection analysis of the yale global tic severity scale. *J Child Adolesc Psychopharmacol.* (2011) 21:621–7. 10.1089/CAP.2010.0149 22070181 PMC3279714

[33] Nissen JB Kaergaard M Laursen L Parner E Thomsen PH. Combined habit reversal training and exposure response prevention in a group setting compared to individual training: a randomized controlled clinical trial. *Eur Child Adolesc Psychiatry.* (2019) 28:57–68. 10.1007/s00787-018-1187-z 29956034 PMC6349803

[34] Becker A Roessner V Breuer D Döpfner M Rothenberger A. Relationship between quality of life and psychopathological profile: data from an observational study in children with ADHD. *Eur Child Adolesc Psychiatry.* (2011) 20:267–75. 10.1007/S00787-011-0204-2/TABLES/5 PMC3180591 21901415

[35] Ricketts EJ Goetz AR Capriotti MR Bauer CC Brei NG Himle MB A randomized waitlist-controlled pilot trial of voice over Internet protocol-delivered behavior therapy for youth with chronic tic disorders. *J Telemed Telecare.* (2016) 22:153–62. 10.1177/1357633X15593192 26169350 PMC6033263

